# Transmembrane TM3b of Mechanosensitive Channel MscS Interacts With Cytoplasmic Domain Cyto-Helix

**DOI:** 10.3389/fphys.2018.01389

**Published:** 2018-10-01

**Authors:** Xiaomin Wang, Siyang Tang, Xiaoxu Wen, Lang Hong, Feifan Hong, Yuezhou Li

**Affiliations:** Children’s Hospital and Department of Biophysics, National Health Commission and Chinese Academy of Medical Sciences Key Laboratory of Medical Neurobiology, Zhejiang University School of Medicine, Hangzhou, China

**Keywords:** mechanosensitive channel, domain interaction, mechanotransduction, ion channel, channel activation, transmembrane domain, membrane protein

## Abstract

The mechanosensitive channel MscS functions as an osmolyte emergency release-valve in the event of a sudden decrease in external environmental osmolarity. MscS has served as a paradigm for studying how channel proteins detect and respond to mechanical stimuli. However, the inter-domain interactions and structural rearrangements that occur in the MscS gating process remain largely unknown. Here, we determined the interactions between the transmembrane domain and cytoplasmic domain of MscS. Using *in vivo* cellular viability, single-channel electrophysiological recording, and cysteine disulfide trapping, we demonstrated that N117 of the TM3b helix and N167 of the Cyto-helix are critical residues that function at the TM3b-Cyto helix interface. *In vivo* downshock assays showed that double cysteine substitution at N117 and N167 failed to rescue the osmotic-lysis phenotype of cells in acute osmotic downshock. Single-channel recordings demonstrated that cysteine cross-linking of N117C and N167C led to a non-conductive channel. Consistently, coordination of the histidines of N117H and N167H caused a decrease in channel gating. Moreover, cross-linked N117 and N167 altered the gating of the severe gain-of-function mutant L109S. Our results demonstrate that N117–N167 interactions stabilize the inactivation state by an association of TM3b segments with β-domains of the cytoplasmic region, providing further insights into the gating mechanism of the MscS channel.

## Introduction

The ability to sense and respond to mechanical stimuli is essential for all life (Gillespie and Walker, 2001; Hamill and Martinac, 2001). Mechanosensitive channels that detect and convert external mechanical forces into intracellular electrical or chemical signals play an important role in mechanotransduction (Anishkin and Kung, 2005; Arnadottir and Chalfie, 2010). Mechanosensitive channels have been implicated in diverse physiological processes, including hearing, touch, pain sensation, and blood pressure control (Delmas et al., 2011; Ranade et al., 2015). Due to their tractable nature, bacterial mechanosensitive channels have served as excellent models for the study of how channels gate in response to mechanical forces (Kung et al., 2010; Booth and Blount, 2012).

**FIGURE 1:**
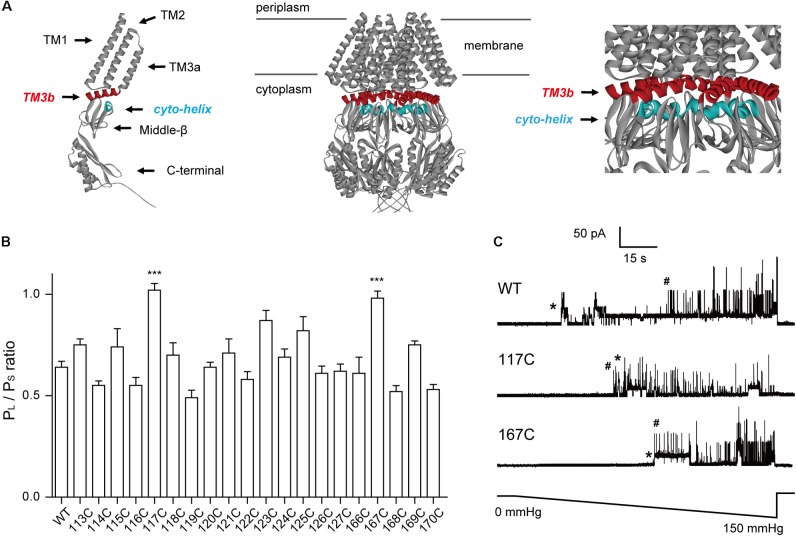
Introduction of cysteine residues into TM3b and the Cyto-helix alters the gating characteristics. **(A)** Schematic of the MscS channel from *E. coli* (PDB code 2OAU). Left: side view of an individual MscS subunit with the positions of the transmembrane helices (TM1, TM2, TM3a, and TM3b). The Cyto-helix, middle-β, and C-terminal are labeled. Middle: side view of the MscS heptamer. Right: enlarged side view of the TM3b and Cyto-helix region. Red, TM3b; blue, Cyto-helix. **(B)** Thresholds for MscS gating as determined by patch clamp (mean ± SEM, *n* = 6; ^∗∗∗^*p* < 0.001 vs. WT MscS). Single-channel recordings and gating threshold measurements were performed using strain MJF429 expressing MscS channels. **(C)** Representative single-channel activity traces for WT, N117C, and N167C. Lower trace: negative pressure application. ^∗^MscS activity; ^#^MscL activity.

The *Escherichia coli* mechanosensitive channel of small conductance, MscS, acts as an *in vivo* emergency release-valve that prevents bacteria from lysis upon an osmotic downshift by releasing osmolytes (Levina et al., 1999; Sukharev, 2002). MscS forms a homoheptamer, each subunit being composed of three transmembrane helices (TM1, TM2, and TM3) and a large cytoplasmic region (Bass et al., 2002; Wang et al., 2008; Lai et al., 2013) (**Figure 1A**). The pore of MscS is formed by the packing of TM3s, which may be divided into two helical segments, TM3a and TM3b, separated by a kink or hinge at Gly113 (Edwards et al., 2005). The first resolved crystal structure of wild-type (WT) MscS (PDB code 2OAU) suggested that its conformation resembles a non-conductive inactivated state (Bass et al., 2002). The other structure for the A106V variant (PDB code 2VV5) is believed to represent an open or partially open state (Wang et al., 2008). A more recent crystal structure of the WT (PDB code 4HWA) solved in dodecylmaltoside shows a partially open state, indicating that the conformation of MscS is sensitive to the type of detergent (Lai et al., 2013). According to the crystal structures, significant rearrangements occur in the TM helices. These include the separation of the TM1–TM2 hairpins that accompany a pivoting of the TM3a helix around Gly113, which lead to opening of the channel pore. Modeling studies have proposed a three-state scheme of the functional MscS cycle: closed, open, and inactivated states (Akitake et al., 2005; Anishkin et al., 2008a,b; Vasquez et al., 2008). The emerging model shows that straightening and buckling of the TM3b define the gating cycle of MscS. Closure and desensitization rely on buckling near Gly121, while the kink at Gly113 is a feature of the inactivated state (Akitake et al., 2007; Belyy et al., 2010).

The cytoplasmic region, which consists of middle-β and COOH-terminal domains, forms a large chamber that has some orifice or wholes or windows but “it is not perforated.” The cytoplasmic region has been suggested to play a role in regulating channel opening (Cox et al., 2013) and to serve as an ion-selective filter (Edwards et al., 2008; Gamini et al., 2011; Zhang et al., 2012). Despite the similarities in the cytoplasmic region of solved structures, there are indications of structural coupling of the cytoplasmic β-domain and TMs (Sotomayor and Schulten, 2004; Nomura et al., 2008; Machiyama et al., 2009; Koprowski et al., 2011; Rowe et al., 2014). The salt bridge formation between D62 in the loop that connects TM1 and TM2, and R131 on the upper face of the β-domain, has been found in molecular dynamics simulations and the functional role of this electrostatic interaction has been experimentally demonstrated to affect the channel inactivation rate (Nomura et al., 2008). Subsequent genetic screens have isolated many mutants in TM3 and the β-domain affecting channel gating and the bacterial growth phenotype. One mutation at the interface between the TM3 and β-domain (G168D) strongly affects the process of adaptation, suggesting that a TM3-cytoplasmic interaction stabilizes the inactivated state in MscS (Koprowski et al., 2011). Recently, the TM3b-β interface has been demonstrated to be involved in sensing macromolecular crowding (Rowe et al., 2014). This study indicated that the coupling of the gate formed by TM3 helices to the peripheral TM1–TM2 pairs is dependent on the state of the association of TM3b with the β-domain. These findings suggest that the interactions between the TMs and cytoplasmic β-domain play a significant role in MscS gating.

The β-domain contains five strands that pack together forming a barrel-like β-sheet around the protein. A small helix (Cyto-helix, residues 166–171) is located on the upper surface of the β-domain and is spatially adjacent to TM3b of MscS (**Figure 1A**). In this study, we set out to investigate the interaction between the TM3b and Cyto-helix. In this study, we have investigated the interaction between the TM3b and Cyto-helix. We generated a mutant library in which every amino acid in the TM3b helix and Cyto-helix had been sequentially replaced with cysteine. Utilizing *in vivo* cellular viability and electrophysiological single channel recording, we found that N117 in the TM3b helix and N167 in the Cyto-helix are critical for mechanosensitive channel normal gating. Disulfide and zinc cross-linking of N117–N167 was found to significantly decrease the pressure-induced current of the double cysteine mutant N117C/N167C and histidine mutant N117H/N167H MscS. Our results suggest that N117 and N167 are key residues involved in the interaction between the transmembrane domain and cytoplasmic domain.

## Materials and Methods

### Strains and Cell Growth

*E. coli* strains MJF465 (Δ*mscL::Cm*, Δ*mscS*, Δ*mscK::Kan*) and MJF429 (Δ*mscS*, Δ*mscK::Kan*) were used to express MscS constructs (Levina et al., 1999; Edwards et al., 2005). Viability assays, cysteine-trapping, and most of the electrophysiological recordings were performed in MJF465, and gating thresholds were determined in MJF429. *E. coli* strain DH10β was used for site-directed mutagenesis. All strains were stored in glycerol stock solution (65% glycerol, 0.1 M MgSO_4_, 0.025 M Tris-Cl) at -80°C. The strains were grown in a Luria-Bertani (LB) medium containing (in g/L) 5 NaCl, 5 yeast extract, and 10 tryptone. The solid LB medium contained 15 g/L agar. Ampicillin was added at 100 μg/ml where appropriate. To initiate experiments, we streaked fresh LB agar plates or LB-ampicillin agar plates with the desired strain, and cells were grown overnight at 37°C. Expression of MscS protein was induced by addition of 1 mM isopropyl-β-D-thiogalactopyranoside (IPTG).

### Constructions of Mutants

*E. coli* MscS without 6 × His-tag (Ec-MscS) was cloned into pB10b, which has an ampicillin-resistance gene and the lacUV5 promotor, as described previously (Zhang et al., 2012). pB10b-Ec-MscS was used as the template in site-directed mutagenesis PCR reactions with the corresponding primers. Successful mutagenesis was verified by DNA sequencing of both strands. Constructs were transformed into MJF465 or MJF429. In Western blot experiments, a 6 × His-Tag was added to the C-terminus of MscS for anti-His antibody recognition.

### Growth Phenotype of Gain-of-Function (GOF) Mutants

Single colonies of *E. coli* MJF465 transformed with MscS constructs were inoculated into LB broth containing 100 μg/ml ampicillin and incubated overnight at 37°C with shaking. The fresh overnight cultures were diluted (1:200) in LB-ampicillin broth, and a subsequent OD_600_ was taken every 30 min. When the OD_600_ was ∼0.2, cultures were equally divided into two 15-ml tubes. Cultures in one tube were not induced, and cultures in the other tube were induced by addition of 1 mM IPTG. All cultures were further incubated at 37°C with shaking, and an OD_600_ was measured every 30 min. All growth experiments were performed in triplicates.

### Viability Assay

All survival experiments were performed using transformants of MJF465 as previously described (Bartlett et al., 2004; Yoshimura et al., 2004). The bacteria were grown in liquid LB broth plus 100 μg/ml ampicillin overnight at 37°C with shaking at 250 rpm. The fresh overnight cultures were diluted (1:100) in 1 ml LB-ampicillin medium and grown for 1 h. The cultures were then diluted (1:1) in 1 ml of the same LB-ampicillin medium supplemented with 1 M NaCl and incubated with shaking. When the OD_600_ was ∼0.2, the cultures were induced with 1 mM IPTG for 1 h. The induced cultures were then diluted 1:20 in LB medium supplemented with 0.5 M NaCl (mock shock) or in distilled water (osmotic downshock). Cells were grown with shaking at 37°C for 20 min. Six consecutive 1:10 serial dilutions were made in LB containing either 0.5 M NaCl or no salt. 2.5 μl of the diluted cells was dropped onto LB-ampicillin agar plates and grown overnight at 37°C. The colonies were counted for viability analysis. Downshock viability was calculated by dividing the colony numbers in the osmotic downshock medium with the colony numbers in the mock shock medium.

### Cysteine Trapping and Western Blots

As described previously (Iscla et al., 2007), cultures grown in the LB medium containing 0.5 M NaCl were induced with 1 mM IPTG for 1 h, and then diluted 1:20 in LB, or LB with 500 uM copper-phenanthroline. Cells were continually grown at 37°C for 20 min. Samples were immediately spun down at 5,000 rpm for 10 min and washed several times. The pellet was then resuspended with non-reducing SDS-PAGE loading buffer and boiled at 85°C for 10 min. For the treatment of βME, cells diluted in LB with 500 μM copper-phenanthroline, and then spun down and resuspended with loading buffer with 20 mM βME. Proteins were separated in 12% SDS-PAGE gels and electrotransferred to a PVDF membrane at 300 pA for 45 min. The membrane was then blocked, by incubation in 2% non-fat powdered milk in TBS buffer, for 1 h. After blocking, MscS-His6 was binding by incubation with monoclonal anti-His antibodies overnight at 4°C reducing. After washing, the secondary antibody, Donkey anti-Rabbit, was added and incubated at room temperature for 2 h.

### Preparation of Spheroplasts

*E. coli* spheroplasts from MJF465 and MJF429 containing MscS constructs were prepared as previously described (Blount et al., 1996; Ou et al., 1998). Single colonies were inoculated into 1 ml LB broth containing 100 μg/ml ampicillin and grown overnight at 37°C in a shaking rotator. The overnight cultures were diluted (1:100) in fresh LB broth containing 100 μg/ml ampicillin, and then incubated at 37°C with shaking. When the OD_600_ was ∼0.2, the cultures were diluted (1:10) in fresh LB broth containing 100 μg/ml ampicillin and 60 μg/ml cephalexin and incubated until 50–150 μm filamentous bacteria became apparent. Subsequently, 1 mM IPTG was added to induce the expression of MscS. After the cultures were induced for 40 min, bacteria were spun down at 2,000 × *g* for 10 min and the pellets were gently re-suspended in 2.5 ml 0.8 M sucrose. Then the cells were digested by adding 125 μl of 1 M Tris-Cl pH 8.0, 30 μl of 5 mg/ml lysozyme, 30 μl of 5 mg/ml DNase, and 30 μl of 125 mM Na-EDTA pH 7.8 in turn. After 5 min incubation, the reaction was terminated by the addition of 1 ml stop solution (875 μl of 0.8 M sucrose, 125 μl ddH_2_O, 20 μl of 1 M MgCl_2_, and 10 μl of 1 M Tris-Cl). The mixture was subsequently placed on ice and diluted in 10 ml of dilution solution (10 ml of 0.8 M sucrose, 100 μl of 1 M MgCl_2_, and 100 μl of 1 M Tris-Cl) in 15-ml tubes. Spheroplasts were spun down at 1,800 × *g* for 5 min. The supernatant was removed and the pellet re-suspended in dilution solution. Spheroplasts were stored in 25-μl aliquots at -80°C.

### Electrophysiology Analysis

Patch clamp recordings were performed on giant *E. coli* spheroplasts as described previously (Sukharev, 2002; Edwards et al., 2005). Excised, inside-out patches were examined at a membrane potential of -20 mV under symmetrical conditions with bath and pipette solutions consisting of (in mM) 200 KCl, 90 MgCl_2_, 10 CaCl_2_, and 5 HEPES. The pH of the patch buffer was adjusted to 6.0, except for recordings from N117H MscS, N167H MscS, and N117H/N167H MscS, which had to be treated with ZnCl_2_ at pH 8.0 for histidine coordination. ZnCl_2_ (2 mM) was added to the bath and left for 5–20 min for this purpose. To cleave the disulfide bridges formed in the MscS cysteine mutants N117C/N167C, 10 mM β-mercaptoethanol (βME) was added to the bath and left for 10–30 min. To enhance the formation of disulfide bridges, 2–20 μM CuII was added to the bath and left for 10–30 min. All data were acquired at a sampling rate of 20 kHz with a 5-kHz filter using an AxoPatch 200B amplifier in conjunction with Clampex (Molecular Devices, San Jose, CA, United States). Negative pressure was applied through the recording electrode using a High Speed Pressure Clamp (ALA Scientific Instruments, Farmingdale, NY, United States). The timing and intensity of the pressure were controlled by signals generated by Clampex. The mechanosensitivity of *E. coli* MscS mutants was tested in the MJF429 strain. To normalize for differences in the pressure required to open channels due to patch-to-patch variations, Mechanosensitive channel of large conductance (MscL) was used as an internal standard in the MJF429 strain. MscL and MscS sense membrane tension and are affected equally by variations in patch geometry. The pressure threshold for activation of the MscS channel was referenced against the activation threshold of MscL to determine the pressure ratio for gating (*P*_S_/*P*_L_). *P*_S_/*P*_L_ ratios were calculated from at least three patches from at least two independent spheroplast preparations. Measurements were analyzed using Clampfit 10.2 (Molecular Devices, San Jose, CA, United States).

## Results

### Cysteine Mutations in TM3b and the Cyto-Helix Alter the Mechanosensitivity of the MscS Channel

Cysteine scanning has been used to determine functional regions within transmembrane domains (Bartlett et al., 2004; Levin and Blount, 2004; Iscla et al., 2007). Here, we used this approach to identify the crucial residues in the TM3b-Cyto helix interface. Due to its relatively small size and near mid-range hydrophobicity, cysteine substitution is unlikely to distort the global structure. Hence, changes in a gated channel suggest that the substituted residue is important for normal channel function. In addition, cysteine may have the advantage of forming disulfide bridges, thus serving to determine the proximity of residues in the protein. Moreover, the absence of endogenous cysteine residues in *E. coli* MscS makes each substituted cysteine unique. In this study, we generated single cysteine-substitution mutants spanning the TM3b helix (residues 113–127) and the Cyto-helix (residues 166–171). Western blots indicated that the expression of single cysteine mutant protein was similar to that of WT MscS (**Supplementary Figure S1**). The channel activity of MscS mutants was then assessed by applying negative pressure to the inside-out patches of giant spheroplasts from the MJF429 strain (null for *MscS* and *MscK*). This strain had two advantages for the study: first, the single cysteine MscS mutants were studied in a background devoid of MscS and MscS-like channel activity; and second, the presence of endogenous MscL activity provided an internal standard to gauge the pressure-sensitivity of MscS mutants. The ratio of the pressure needed to open MscS channels, relative to that required for control MscL channels, which were present in the same patch membrane, was defined as the MscS gating threshold (*P*_S_/*P*_L_) (Miller et al., 2003; Nomura et al., 2006). Consistent with the results from previous studies (Sotomayor and Schulten, 2004; Zhang et al., 2012), a gating threshold of 0.64 ± 0.11 (*n* = 6) was determined for WT MscS (**Figures 1B,C**). The gating threshold of most cysteine-substituted mutants did not differ from that of the WT, except for the N117C and N167C mutants. The sensitivity to pressure was significantly reduced in N117C and N167C (*P*_s_/*P*_L_ = 1.02 ± 0.08 and 0.97 ± 0.09, respectively, *n* = 6), both of which gated at a pressure close to that required for MscL (**Figure 1C**). These results demonstrated that N117 and N167 are the important residues in TM3b and the Cyto-helix.

### Cysteine Substitutions at N117 and N167 Affect MscS Channel Gating

**FIGURE 2:**
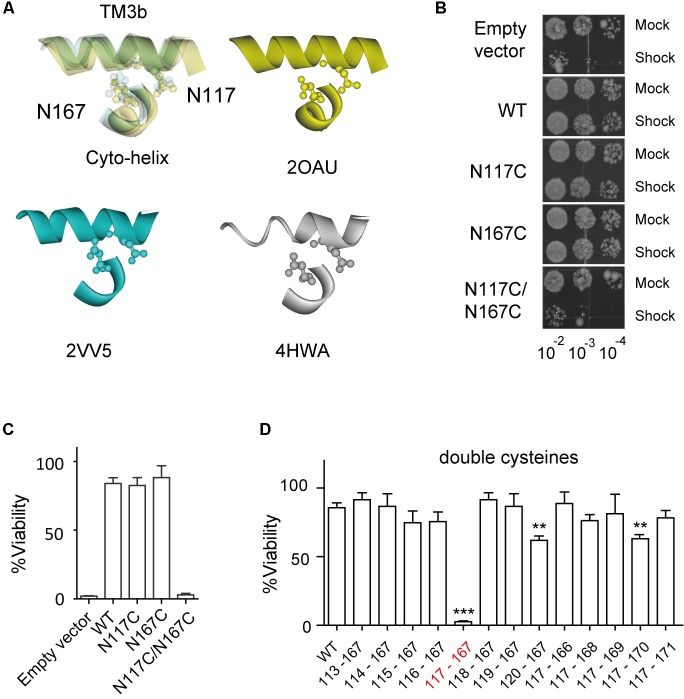
Double substitutions of N117C and N167C cause an LOF phenotype. **(A)** Enlarged superimposing view of N117 in TM3b and N167 in Cyto-helix depicted in a space-filling model from three crystal structures (PDB codes 2OAU, 2VV5, and 4HWA). Yellow, 2OAU; blue, 2VV5; gray, 4HWA. **(B)** Growth of the MJF465 (Δ*mscl*, Δ*mscs*, Δ*msck*) strain harboring empty vector or MscS. The panels show the 5-μl drop-plates at each tenfold dilution (10^-2^ to 10^-4^) of cells treated with mock shock (Mock) or osmotic downshock (Shock). **(C,D)** Percentage viability of cells exposed to osmotic downshock relative to mock shock (mean ± SEM, *n* = 5). ^∗∗^*p* < 0.01, ^∗∗∗^*p* < 0.001 vs. WT MscS.

The crystal structure of *E. coli* MscS, solved from either the inactivated or the open state, reveals that N167 of the Cyto-helix is packed closely with N117 of TM3b (**Figure 2A**), suggesting a potential interaction between them. To determine whether N167 interacts with N117, a double cysteine mutant (N117C/N167C) was generated and its effects on channel function were assessed using an *in vivo* downshock assay. MscS proteins were expressed in strain MJF465 (*mscL^-^*, *mscS^-^*, *mscK^-^*), which has none of the known mechanosensitive channels (MscL, MscS, and MscK). Just like WT MscS, the expression of either the N117C or the N167C single mutant rescued the osmotic-lysis phenotype of MJF465 on acute osmotic downshock (**Figures 2B,C**). In contrast, the N117C/N167C double mutant did not rescue growth and its viability was extremely low (2.54 ± 0.65, *n* = 5), more like an empty vector (1.78 ± 0.47, *n* = 5). Interestingly, most of other double mutants with cysteine substitutions at N167 and one neighboring residue of N117 in TM3b (G113C-A120C/N167C) showed viability comparable to the WT (**Figure 2D**). Similar results were obtained for the double mutants with cysteine substitutions at N117 and one neighboring residue of N167 in the Cyto-helix (N117C/P166C-I171C). It is noteworthy that A120C/N117C and N117C/N170C showed the significant reduced viability (60.89 ± 3.75, 63.09 ± 2.25, respectively, *n* = 5) than that of WT (85.63 ± 7.90, *n* = 5), suggesting the potential interaction between the residues. However, both of A120C/N117C and N117C/N170C still rescued more than 60% of the osmotic-lysis phenotype, in contrast to none of viability of N117C/N167C under acute osmotic downshock. Thus, cysteine substitution at only N117 and N167C leads to loss-of-function (LOF) of the MscS channel.

**FIGURE 3:**
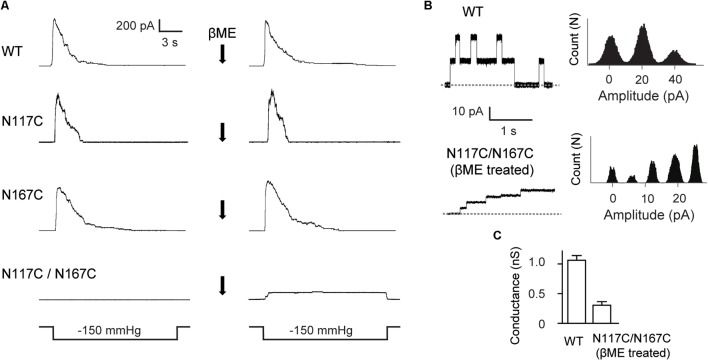
Double substitutions of N117C and N167C inhibit the gating of MscS. **(A)** Representative patch clamp recordings from the same patch before and after addition of βME to the bath. Lower traces: applied negative pressure. MscS expressed in *E. coli* MJF465 strain. **(B)** Left: single-channel openings recorded from WT MscS and βME treated N117C/N167C (dashed horizontal lines indicate closed states); right: count vs. amplitude histograms. **(C)** Conductance of WT MscS and βME treated N117C/N167C determined at 20 mV membrane potential (mean ± SEM, *n* = 6).

We then made single-channel recordings to further investigate the influence of cysteine substitution at N117 and N167 on MscS channel activity. Channel activity of the double mutant N117C/N167C MscS was not elicited in the excised membrane patches examined (**Figure 3A**). However, channel activity of N117C/N167C became detectable after treatment with the reducing agent βME (20 mM, 20 min), and the channel showed a lack of adaptation once opened. Noted that βME treatment had no effect on the channel activity of WT MscS as well as the N117C and N167C mutants. Together, these results demonstrated that a disulfide bond forms spontaneously between N117C and N167C under non-reducing conditions, thus locking the channel into a non-conductive state.

Although gated under reducing conditions and showing nearly the similar ladder-like pattern of opening as the WT, the openings of N117C/N167C after βME treatment were predominantly occupied at low-conducting states. The single-channel current amplitude of βME treated N117C/N167C was much lower than the WT, and the single-channel conductance was 0.26 ± 0.07 nS (*n* = 6), significantly lower than that of the WT (∼1 nS) (**Figures 3B,C**).

### Coordination of Histidines by Zn^2+^ Decreases Channel Activity of Double-Mutant (N117H/N167H) MscS

**FIGURE 4:**
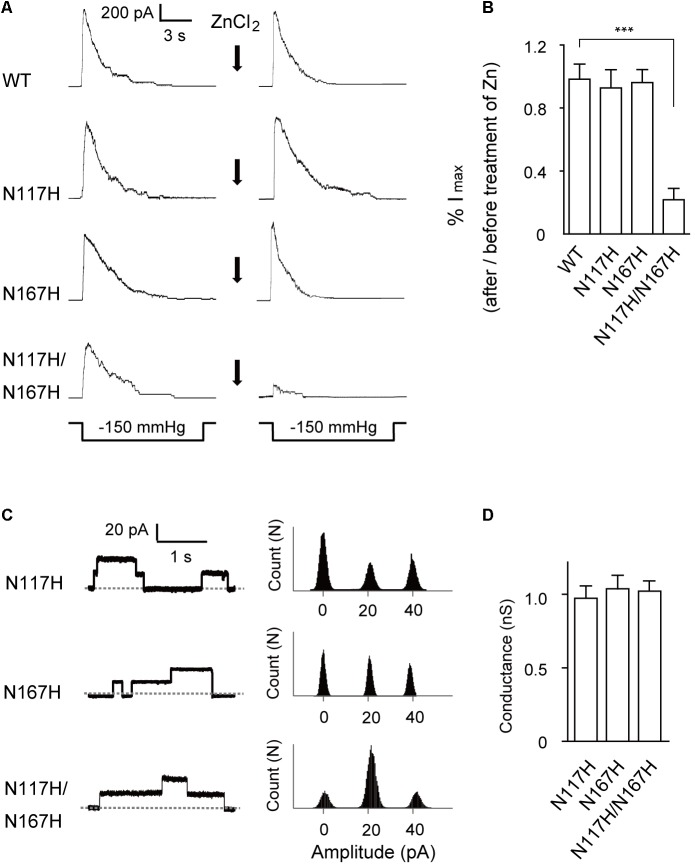
Activation of N117H/N167H MscS is decreased by adding ZnCl_2_. **(A)** Representative patch clamp recordings from the same patch before and after addition of 1 mM ZnCl_2_ to the bath. The recordings are from the *E. coli* MJF465 strain; lower traces: applied negative pressure. Channels without His-tag were used in this experiment. **(B)** Pressure-activated current significantly decreased in N117H/N167H MscS with ZnCl_2_ treatment (mean ± SEM, *n* = 6; ^∗∗∗^*p* < 0.001). **(C)** Left: single-channel openings recorded from histidine substitutions after ZnCl_2_ treatment (dashed horizontal lines indicate closed states); right: count vs. amplitude histograms. **(D)** Conductance of histidine substitutions after ZnCl_2_ treatment determined at 20 mV membrane potential (mean ± SEM, *n* = 6).

Next, we used a Zn^2+^ cross-linking method to further investigate the effects of N117–N167 interactions on channel activation. Given that the presence of Zn^2+^ could coordinate histidine residues within the complex and therefore lead to what was effectively cross-linking of the specific mutated sites (Iscla et al., 2008), the double-histidine mutant N117H/N167H was assessed for changes in channel activity under Zn^2+^ treatment. The maximal currents in response to an identical stimulus protocol and recorded from the same patch before and after treatment with ZnCl_2_ were compared. WT MscS and the N117H or N167H single mutant did not significantly differ after ZnCl_2_ treatment (**Figure 4A**). In comparison, only 21 ± 7.62% (*n* = 6) N117H/N167H MscS current was activated in response to the same pressure stimulus after exposure to ZnCl_2_ for 10 min (**Figure 4B**). Moreover, the single-channel amplitude analysis of histidine substitutions after ZnCl_2_ treatment indicated that coordination of histidine did not decrease the single-channel conductance (**Figures 4C,D**). Thus, cross-linking of N117H to N167H effectively inhibit the opening of MscS.

### Cysteine Substitutions at N117 and N167 Alter the Gating of the GOF Mutant L109S

**FIGURE 5:**
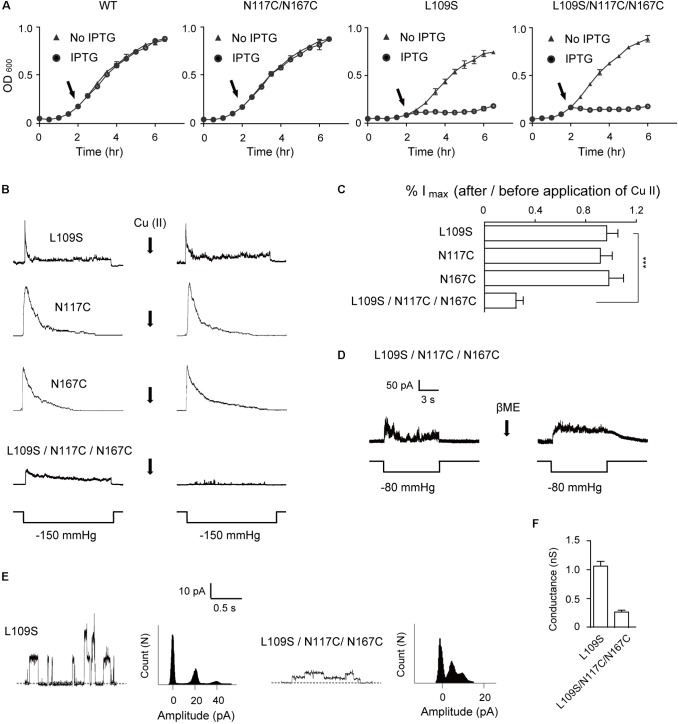
Gating alteration in the triple mutant L109S/N117C/N167C MscS. **(A)** Growth curves of MJF465 cells expressing WT, N117C/N167C, L109S, and L109S/N117C/N167C MscS induced with IPTG (circles) or without induction (triangles). Arrows indicate induction by 1 mM IPTG. **(B)** Representative single-channel recordings of MscS expressed in the *E. coli* MJF465 strain, from the same patch before and after treatment with copper phenanthroline [Cu(II)]. Lower traces: applied negative pressure. **(C)** Analysis of maximum current amplitude before and after treatment with Cu(II) (mean ± SEM, *n* = 6). **(D)** Representative single-channel recordings of L109S/N117C/N167C, from the same patch before and after treatment with βME. Lower traces: applied negative pressure. **(E)** Single-channel openings of L109S and L109S/N117C/N167C MscS (left; dashed horizontal lines indicate the closed state) and count vs. amplitude histograms (right). **(F)** Conductance of L109S and L109S/N117C/N167C MscS determined at 20 mV membrane potential (mean ± SEM, *n* = 6).

It has been shown that L109S is gated at a lower threshold and thus exhibits a severe gain-of-function (GOF) phenotype (Edwards et al., 2005). As revealed by the crystal structure, L109 is located on TM3a and forms a constriction point on the cytoplasmic side of the channel pore. Substitution of leucine for the more hydrophilic serine may break the vapor-lock mechanism, pre-hydrate the closed/inactivated gate and make it leaky. To further investigate the role of N117 and N167 in channel activation, we introduced L109S into N117C/N167C and assessed the function of L109S/N117C/N167C. We expected that the gating of MscS could be retained by L109S to reverse the non-conductive state of N117C/N167C. *In vivo* physiological studies showed that the expression of WT and N117C/N167C did not impair the growth of MJF465 cells with or without IPTG induction (**Figure 5A**), while the mutants L109S and L109S/N117C/N167C caused decreased cell growth upon induction with IPTG, suggesting that L109S/N117C/N167C is a GOF mutant and is thus deleterious to cell growth.

In an attempt to test the hypothesis that the disulfide bridges formed between N117C and N167C affect L109S/N117C/N167C gating, we used copper phenanthroline to promote cysteine cross-linking and examined the changes in channel activity. Patch-clamp recordings showed comparable gating thresholds of L109S (2.12 ± 0.09, *n* = 6) and L109S/N117C/N167C (2.02 ± 0.08, *n* = 6), which is consistent with the GOF phenotype of the cell growth. However, the copper phenanthroline treatment significantly decreased L109S/N117C/N167C current (**Figure 5B**), while had no effect on the activity of single mutants of N117C, N167C, or L109S. The same pressure protocol gated only 24 ± 4.76% of the L109S/N117C/N167C MscS current compared to that before treatment (**Figure 5C**). On the contrary, broken the cysteine cross-linking by βME prohibited the inactivation, and L109S/N117C/N167C showed sustained current even the pressure was released (**Figure 5D**). In addition, the single-channel opening amplitude of L109S was similar to that of the WT (**Figure 5E**), but the triple mutant L109S/N117C/N167C exhibited a significantly decreased conductance of ∼0.25 nS (**Figure 5F**). These results are consistent with the findings for the N117C/N167C double mutant.

### Cysteine Substitutions at N117 and N167 Cause Cross-Linked Multimers

**FIGURE 6:**
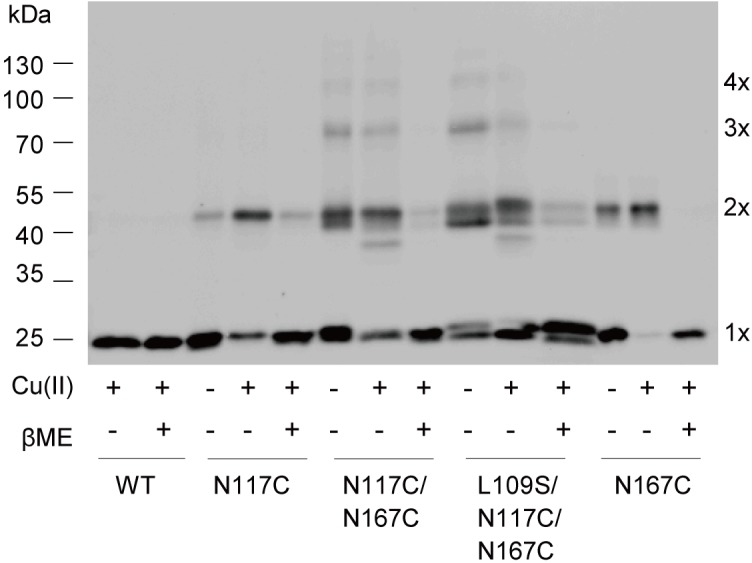
Disulfide bridges formed between N117C and N167C. Western blot showing the cross-linked multimers. Cells expressing WT, N117C, N167C, N117C/N167C, and L109S/N117/N167C, respectively, were treated with 500 μM copper-phenanthroline [Cu(II)] only, or treated with Cu(II) firstly and then with 20 mM βME.

To further explore the details of disulfide bridges formed in double cysteine mutant N117C/N167C MscS gating, we performed *in vivo* cysteine cross-linking experiments as previously described (Iscla et al., 2007). Disulfide trapping is a popular tool for determining cysteines that approach or interact upon channel gating, in which differing band mobility tells us the form of the various multimers. As shown in **Figure 6**, only monomers were present in WT MscS because it contains no endogenous cysteine. N117C/N167C and L109S/N117C/N167C MscS showed the obvious multimer bands. Noted that these bonds indeed formed between disulfide bridge from cysteines, since the multiple bonds can be reduced reversibly by βME.

## Discussion

In this study, we investigated the interactions between the TM3b and Cyto-helix domain of the MscS channel. The crystal structure and genetic screens revealed that TM3b and the Cyto-helix were spatially close and several substitutions within the domains were found to affect channel gating and the bacterial growth phenotype. However, it was unclear which residues were most functionally important in the TM3b-Cyto-helix interface. Using a cysteine substitution scanning method, we demonstrated that N117C in TM3b and N167C in the Cyto-helix exhibited significant increases in the gating threshold. N117C and N167C double substitutions led to a LOF phenotype as determined by *in vivo* downshock assays, whereas other double mutants within the TM3b-Cyto-helix interface presented a growth phenotype similar to that of WT MscS. Subsequent single-channel recordings confirmed the LOF phenotype of N117C/N167C, and demonstrated that negative pressure did not activate the double mutant unless the reducing agent βME was present. These results demonstrated the efficient formation of disulfide bridges in N117C and N167C in the TM3b-Cyto-helix interface, and that the disulfide cross-linking of N117C and N167C caused the loss of channel activity.

Metal binding to endogenous and substituted histidines has been used to elucidate the mechanisms of MscS and MscL gating. Coordination of the histidines in the MscS C-terminal by Ni^2+^ results in a decrease in MscS channel activity, implying that the C termini move apart upon channel gating (Koprowski and Kubalski, 2003). Another study has shown that the addition of Zn^2+^ leads to a decrease in conductance to A110H and A112H in MscL (Yang et al., 2012). Here, we showed that the coordination of histidines by Zn^2+^ resulted in a decreased MscS current in the N117H/N167H double mutant, but not in the N117H or N167H single mutant, supporting the conclusion that tight interactions of N117 and N167 inhibit MscS channel activation.

Our findings indicate that N117C and N167C in the TM3b-Cyto-helix interface approach closely as the channels undergoes gating. The double mutant N117C/N167C exhibited an LOF phenotype and disulfide bridges of N117C and N167C may lock the association of TM3b and the Cyto-helix. But we don’t have evidence to discriminate whether the mutant channels go directly to the inactivated state or simply stabilize a closed state and never open. Recently genetic screens for potassium leaky indicated that N117K and G168D at TM3b-Cyto-helix interface strongly delay the process of inactivation, because the charged mutations weaken the hydrophobic interaction between these two domains (Koprowski et al., 2011). Conversely, hydrophobic substitutions of N117V and N167V stabilize the interactions at the TM3b-Cyto-helix interface, and exhibit a rapidly inactivated channel activity (Rowe et al., 2014). Cross-linked N117C/N167C may gate at a considerably higher tension than the WT, produce a highly unstable open state, and prefer to enter into the inactivated state without opening, which leads to the LOF phenotype and undetectable channel activity.

Although the tight cross-linking of N117 and N167 precluded opening, we detected channel opening under reduced conditions. However, both L109S/N117C/N167C and βME treated N117C/N167C showed significantly decreased channel opening amplitude with a conductance of ∼0.25 nS. In addition, once channels opened after βME treatment, they never came back to the closed or the inactivated state. These results indicate the importance of the residues of N117 and N167, and that substitutions of cysteine may change the gating process of the channel.

One of the interesting finding is that the triple mutant L109S/N117C/N167C showed GOF growth similar to that of the L109S mutation, despite the clear LOF phenotype of N117C/N167C. Although low-conducting opening states and cross-linked multimers were found, the interaction of N117C and N167C did not completely inactivate the channel activity of L109S/N117C/N167C in ambient conditions. The fact that the channel activation of L109S/N117C/N167C decreased significantly under oxidative conditions suggests that L109S substitution interferes with the N167C–N167C interaction. Obviously, in the triple mutant, the presence of L109S, either decrease the cross-linking ability of N117C and N167C, or it influences the result of the cross-link, therefore the observed level of current which is different to the non-conducting phenotype shown by the double mutant N117C/N167C. However, at present predictions of the rearrangements of TM3 and the Cyto-helix are difficult to assess without further structural data for the channel.

In summary, our results support the conclusion that the interaction between the transmembrane domain and cytoplasmic domain of MscS plays an important role in the gating process. We demonstrated that N117 on the TM3b helix and N167 on the Cyto-helix approach closely and form efficient interactions. They are key residues in the regulation of MscS gating through Cyto-helix interaction.

## Author Contributions

XW conducted most of the experiments. ST and LH generated the molecular cloning. XW and FH performed the cell growth study. YL supervised the study.

## Conflict of Interest Statement

The authors declare that the research was conducted in the absence of any commercial or financial relationships that could be construed as a potential conflict of interest.
