# Supplementary material for: Transmembrane TM3b of Mechanosensitive Channel MscS Interacts With Cytoplasmic Domain Cyto-Helix
Source: Front Physiol. 2018 Oct 1;9:1389. doi: 10.3389/fphys.2018.01389 (PMC6174206; doi:10.3389/fphys.2018.01389)
Supplement: Supplementary file 1 [file Image_1.pdf]

Supplemental Figures

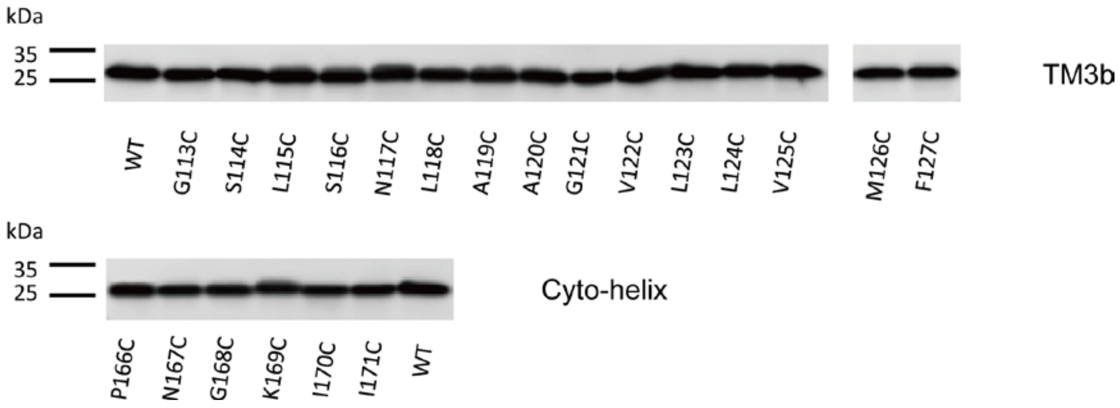

Figure S1 Western blot of *E.coli* MscS channels with cysteine mutation in TM3b and Cyto-helix. Cultures grown in the LB medium were induced with 1mM IPTG for 1 hour. Samples were immediately spun down at 5000rpm for 10min. The pellet was resuspended with non-reducing SDS-PAGE loading buffer and boiled at 85°C for 10min. Proteins were separated in 12% SDS-PAGE gels and electrotransferred to a PVDF membrane at 300 pA for 45min. The membrane was then blocked, by incubation in 2% non-fat powdered milk in TBS buffer, for 1 hour. After blocking, MscS-His6 was binding by incubation with monoclonal anti-His antibody overnight at 4°C reducing. After washing, the secondary antibody, Donkey anti-Rabbit, was added and incubated at room temperature for 2 hours.
